# Comparative oncology: The paradigmatic example of canine and human mast cell neoplasms

**DOI:** 10.1111/vco.12440

**Published:** 2018-09-24

**Authors:** Michael Willmann, Emir Hadzijusufovic, Olivier Hermine, Mauro Dacasto, Laura Marconato, Karin Bauer, Barbara Peter, Susanne Gamperl, Gregor Eisenwort, Erika Jensen‐Jarolim, Mathias Müller, Michel Arock, David M. Vail, Peter Valent

**Affiliations:** ^1^ Department of Companion Animals and Horses, Clinic for Internal Medicine University of Veterinary Medicine Vienna Vienna Austria; ^2^ Ludwig Boltzmann Cluster Oncology Medical University of Vienna Vienna Austria; ^3^ Department of Medicine I Division of Hematology and Hemostaseology, Medical University of Vienna Vienna Austria; ^4^ Department of Hematology, Necker Hospital Imagine Institute Université Paris Descartes, Sorbonne Paris France; ^5^ Department of Comparative Biomedicine and Food Science University of Padua Padua Italy; ^6^ Centro Oncologico Veterinario Sasso Marconi Italy; ^7^ Institute of Pathophysiology and Allergy Research, Center of Pathophysiology, Infectiology and Immunology Medical University of Vienna Vienna Austria; ^8^ The Interuniversity Messerli Research Institute University of Veterinary Medicine Vienna, Medical University Vienna, University of Vienna Vienna Austria; ^9^ Biomodels Austria and Institute of Animal Breeding and Genetics University of Veterinary Medicine Vienna Austria; ^10^ LBPA CNRS UMR8113, Ecole Normale Supérieure de Cachan Cachan France; ^11^ Laboratory of Hematology Pitié‐Salpêtrière Hospital Paris France; ^12^ Department of Medical Sciences, School of Veterinary Medicine University of Wisconsin‐Madison Madison Wisconsin; ^13^ Carbone Cancer Center University of Wisconsin‐Madison Madison Wisconsin

**Keywords:** canine mast cell neoplasm, CD25, CD30, *KIT* mutations, tryptase

## Abstract

In humans, advanced mast cell (MC) neoplasms are rare malignancies with a poor prognosis. Only a few preclinical models are available, and current treatment options are limited. In dogs, MC neoplasms are the most frequent malignant skin tumours. Unlike low‐grade MC neoplasms, high‐grade MC disorders usually have a poor prognosis with short survival. In both species, neoplastic MCs display activating *KIT* mutations, which are considered to contribute to disease evolution. Therefore, tyrosine kinase inhibitors against KIT have been developed. Unfortunately, clinical responses are unpredictable and often transient, which remains a clinical challenge in both species. Therefore, current efforts focus on the development of new improved treatment strategies. The field of comparative oncology may assist in these efforts and accelerate human and canine research regarding diagnosis, prognostication, and novel therapies. In this article, we review the current status of comparative oncology approaches and perspectives in the field of MC neoplasms.

## 1 INTRODUCTION

Mast cell (MC) neoplasms are haematopoietic disorders characterized by uncontrolled expansion and accumulation of neoplastic mast cells (MCs) in various organ systems.^1^, ^2^, ^3^, ^4^, ^5^, ^6^, ^7^ In humans, the most frequently affected organs in systemic mastocytosis (SM) are the skin, bone marrow (BM), liver and spleen. Both indolent and aggressive variants of SM have been described.^1^, ^2^, ^3^, ^4^, ^5^, ^6^, ^7^, ^8^, ^9^, ^10^ Indolent variants include patients with cutaneous mastocytosis (CM) and indolent SM (ISM). Advanced forms of SM can be divided into aggressive SM (ASM), SM with an associated hematologic neoplasm (SM‐AHN) and MC leukaemia (MCL).^1^, ^2^, ^3^, ^4^, ^8^, ^9^, ^10^ MC sarcoma (MCS) is an extremely rare, localized form of advanced mastocytosis in humans. Whereas patients with ISM have an excellent prognosis with normal life‐expectancy,^11^ patients with advanced SM or MCS have a poor prognosis with short survival times (STs).^2^, ^3^, ^4^, ^12^, ^13^ For example, Lim and colleagues reported that patients with ASM have a median ST of 41 months, patients with SM‐AHN have a median ST of 24 months, and MCL patients have a median ST of 2 months.^12^

In the canine system, cutaneous MC tumours (MCTs) are frequently detected and can be divided into less aggressive and more aggressive variants. The aggressive MCT can involve regional lymph nodes and/or visceral organs.^5^, ^6^, ^7^, ^14^ It is worth noting that MCTs in dogs are the most frequent malignant skin tumours.^5^, ^6^, ^7^ Whereas histological low‐grade MCTs have a good‐to‐excellent prognosis, metastasized and/or high‐grade canine MCTs have a poor prognosis with short STs.^5^, ^6^, ^7^

In dogs as well as in humans, it is of considerable importance to establish the correct diagnosis and to define whether the patient has an indolent MC neoplasm or an advanced category of the disease.

During the past few decades, the pathogenesis and molecular mechanisms underlying disease evolution and progression of SM have been analysed. In both species, neoplastic MCs display activating *KIT* mutations that are considered to contribute to disease evolution.^1^, ^2^, ^3^, ^4^, ^15^, ^16^, ^17^, ^18^, ^19^, ^20^ Based on this concept, various tyrosine kinase inhibitors (TKI) directed against KIT have been developed with the hope that these agents can act as disease‐modifying drugs.^21^, ^22^, ^23^, ^24^, ^25^, ^26^, ^27^, ^28^, ^29^, ^30^ Unfortunately, clinical responses are unpredictable and often transient in both species, which remains a clinical challenge in daily practice.^31^, ^32^, ^33^, ^34^, ^35^, ^36^, ^37^, ^38^, ^39^, ^40^ Therefore, current efforts focus on deciphering other molecular pathways involved in the pathogenesis of SM in order to establish new treatment strategies.

Comparative oncology is an emerging field that is based on the assumption that the biochemical processes and pathogenesis contributing to the evolution and progression of spontaneous malignancies in human and animal species are often comparable and that these similarities can be exploited in basic and translational science.^41^, ^42^, ^43^, ^44^ Based on this assumption, many different projects in the field of comparative oncology have been initiated.^44^, ^45^, ^46^ One emerging example with future potential might be research on human and canine MC neoplasms.^47^, ^48^ As mentioned before, these neoplasms have several aspects in common, such as *KIT* mutations and a poor outcome in advanced stages.

There is hope that the field of comparative oncology can assist in our efforts to accelerate human and canine research on MCs and to improve diagnosis, prognostication and, ultimately, therapy in MC neoplasms. However, a number of questions regarding classification, prognostication and therapy of advanced MC disorders in both species remain. For example, it remains to be explored whether the diagnostic criteria and prognostic parameters that have recently been established in human MC neoplasms, can be employed in a similar, analogous way in the canine system.

In the present article, we review the current status of comparative oncology approaches in the field of MC neoplasms, with special focus on diagnosis, prognostication and standard treatment of patients with MC neoplasm in humans and dogs.

## 2 CLASSIFICATION OF MC NEOPLASMS AND MINIMAL DIAGNOSTIC CRITERIA

**Figure 1:**
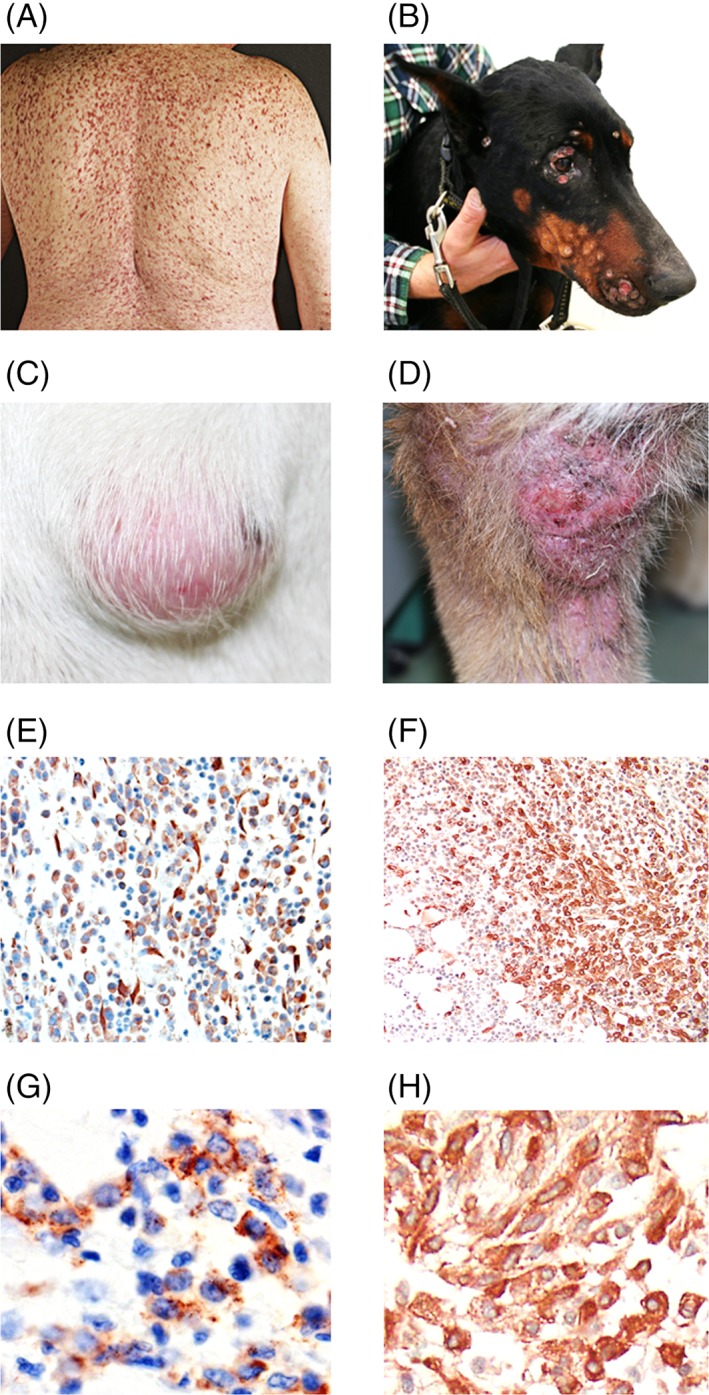
Human and dog mast cell neoplasms. A, Skin involvement in a patient with indolent systemic mastocytosis. B, Disseminated cutaneous lesions of mastocytoma (grade 1 according to the Patnaik Scheme and low‐grade according to the Kiupel Scheme) in a Doberman pinscher. C and D, Localized cutaneous mastocytoma lesions in two different dogs. E, Immunohistochemical detection of neoplastic mast cells in a cutaneous mastocytoma lesion using an antibody against KIT. F, Infiltration of the bone marrow with neoplastic mast cells visualized by tryptase‐staining in a human patient with aggressive systemic mastocytosis. G, KIT‐positive mast cells in a canine patient with multiple cutaneous mastocytoma. H, KIT‐positive mast cells in the bone marrow of human patient with advanced mastocytosis. Original magnifications: E, F: ×200; G, H: ×600 [Colour figure can be viewed at wileyonlinelibrary.com]

Whereas minimal diagnostic criteria for SM have been established in humans, no such criteria have been validated in canine MCT so far. In human SM, the major diagnostic criterion relates to the multi‐focal dense infiltrates of MCs in the BM and other extracutaneous organs.^2^, ^49^, ^50^, ^51^, ^52^, ^53^, ^54^ Minor criteria include the abnormal (often spindle‐shaped) morphology of MC, expression of CD2 and/or CD25 in MCs, KIT‐activating mutations at codon 816 of *KIT* and elevated serum tryptase levels (>20 ng/mL).^2^, ^49^, ^50^, ^51^, ^52^, ^53^, ^54^ When at least one major and one minor or at least three minor criteria are fulfilled, the diagnosis of SM can be established.^2^, ^49^, ^50^, ^51^, ^52^, ^53^, ^54^ At present, it remains unknown whether disease‐related parameters that have already been proven to serve as robust diagnostic parameters in human MC neoplasms, such as abnormal expression of CD25 on neoplastic MCs or certain *KIT* mutations, should undergo validation in dogs with MCTs.^55^, ^56^ In addition, it remains unknown whether elevated serum tryptase levels might serve as diagnostic and follow‐up parameters in dogs with MCTs in the same way as in human MC neoplasms. If one or more of these markers were determined to be of diagnostic value, these parameters could be employed as additional diagnostic criteria in dogs in future proposals. For the moment, the diagnostic tools available to classify canine MCTs are morphological assessments, histochemical studies (such as toluidine blue) and immunohistochemical investigations of neoplastic MCs, which may provide a solid basis for forthcoming comparative oncology studies (Figure 1).^57^, ^58^, ^59^, ^60^

While the diagnosis of canine MCT can be made by a fine‐needle aspiration‐based cytology in a majority of the cases, histological grading of MCT requires histological examinations of the primary tumour site. Occasionally, immunohistochemistry may be necessary to confirm the diagnosis, especially when MCT is undifferentiated. Potential diagnostic criteria for canine MCTs and a comparison with diagnostic criteria for human SM are shown in Table 1.

**Table 1:** Established and potential diagnostic criteria in human and canine mast cell neoplasms

Marker/feature	Employed as diagnostic criterion in	
	Human systemic mastocytosis	Canine mast cell tumour
Typical skin infiltrates	–a	–a
Histology of mast cell infiltrate	+	+
Abnormal morphology of mast cells	+	+
Basal serum tryptase level	+	–
*KIT* mutations	+	–b
Expression of CD2 or CD25 in mast cells	+	–
Expression of CD30 in mast cells	–c	–c

a Typical skin lesions and a positive Darier's sign are diagnostic criteria of human cutaneous mastocytosis and mastocytosis in the skin in those who have systemic mastocytosis. In canines, a positive Darier sign can suggest the presence of a mast cell tumour, but the diagnosis of a mast cell tumour has to be confirmed by cytology and/or histology in all cases.

b The presence of *KIT* mutations (exons 8, 9, and 11) can confirm the presence of a mast cell neoplasm, but is not yet regarded as a disease‐related diagnostic criterion.

c CD30 is aberrantly expressed on neoplastic mast cells and is a new emerging disease‐related parameter and potential new criterion of systemic mastocytosis in humans. Whether CD2, CD25, or CD30 can be employed as diagnostic criteria for canine mast cell tumours remains so far unknown. At least in the human system, CD30 is expected to become a new minor criterion for systemic mastocytosis.

## 3 CLINICAL AND HISTOLOGICAL PRESENTATION OF MC NEOPLASMS

Depending on the affected organ‐system(s), human mastocytosis can be divided into CM, SM, and localized MCTs.^2^, ^49^, ^50^, ^51^, ^52^, ^53^, ^54^ In the human system, the classification of MC neoplasms proposed by the World Health Organization discriminates between several distinct sub‐variants of CM and SM (Table 2). CM is defined by typical features of cutaneous lesions, a diagnostic skin‐histology, and the absence of criteria sufficient to establish the diagnosis SM.^2^, ^8^, ^49^, ^50^, ^51^, ^52^, ^61^ A positive Darier's sign supports the conclusion that the patient is suffering from CM. However, identical cutaneous lesions are also seen in SM (Table 1). Therefore, the lesion is described as mastocytosis in the skin (MIS), and only a BM examination can clarify the final diagnosis (CM or SM) in adults.^2^, ^8^, ^49^, ^50^, ^51^, ^52^, ^61^ It is noteworthy that a minimal infiltration of the BM with neoplastic MCs may be well detected in CM. Even if two minor SM criteria (but no major SM criterion) are found in these patients, the diagnosis remains CM. 2, 8, 49, 50, 51, 52, 61 However, once three minor criteria are documented or at least one major and one minor criteria are found, the final diagnosis is SM.^2^, ^49^, ^50^, ^51^, ^52^ SM variants that have a grave prognosis (SM‐AHN, ASM, and MCL) are also referred to as advanced SM. The major criteria for advanced SM are the documented presence of SM‐induced organ damage, also known as C‐findings (Table 3).^2^, ^49^, ^50^, ^51^, ^52^ Such C‐findings include marked persistent cytopenia, hepatic disease with elevated liver enzymes and ascites, marked osteolyses (with or without pathologic fractures), or malabsorption with hypalbuminemia and weight loss.^2^, ^49^, ^50^, ^51^, ^52^

**Table 2:** Classification systems for human mastocytosis according to the WHO criteria

Variant and sub‐variant(s)	Abbreviation	Minimal diagnostic criteria
Systemic mastocytosis	SM	SM criteria fulfilled^a^: at least one major and one minor or at least three minor SM criteria^a^ (±cutaneous involvement)
Indolent SM	ISM	No C‐finding^b^, <2 B‐findings^b^, no AHN, MCs <20% in BM smears
BM mastocytosis	BMM	Same as ISM but without skin lesions
Smouldering SM	SSM	No C‐finding^b^, 2 or 3 B‐findings^b^, no AHN, MCs <20% in BM smears
SM with an AHN	SM‐AHN	Criteria for SM and for an AHN are fulfilled^c^
Aggressive SM	ASM	One or more C‐findings^b^ and MC <20% on BM smears
Classical ASM	ASM	<5% MCs in BM smears
ASM in transformation	ASM‐t	5%‐19% MCs in BM smears
MC leukaemia	MCL	≥20% MCs in BM smears
Aleukemic MCL		<10% MCs in peripheral blood
Chronic MCL		No C‐finding^b^
Acute MCL		One or more C‐findings^b^
MC sarcoma	MCS	Local aggressive MC tumour, SM criteria not fulfilled
Extracutaneous mastocytoma^d^		Local benign MC tumour, SM criteria not fulfilled

Abbreviations: AHN, associated hematologic neoplasm; BM, bone marrow; MC, mast cell; WHO, World Health Organization.

The classification presented is based on the WHO proposal of 2001,^2^, ^49^ 2008,^50^ and 2017.^52^, ^53^, ^54^ Consensus criteria were first published in 2001,^2^ and were later confirmed by the same group in 2007 and 2012.^51^, ^61^

a Minimal criteria to diagnose SM (SM criteria) are shown in Table 3.

b B‐findings and C‐findings are listed in Table 3.

c The AHN component of disease is classified according to WHO criteria.

d Extracutaneous mastocytomas are exceptionally rare.

**Table 3:** B‐findings and C‐findings recorded in human patients with SM^a^

B‐findings = Indicate a high burden of MCs and expansion of the neoplastic process into multiple haematopoietic lineages without impairment of organ function
Mnemonic: B = borderline benign (be watchful)
1. MC infiltration grade in the BM >30% by histology and basal serum tryptase level > 200 ng/mL
2. Hypercellular BM with loss of fat cells, discrete signs of dysmyelopoiesis without substantial cytopenias or WHO criteria for an MDS or MPN
3. Organomegaly: Palpable hepatomegaly, splenomegaly, or lymphadenopathy (on CT or ultrasound: >2 cm) without impaired organ function
C‐findings = Indicate organ damage produced by MC infiltration (should be confirmed by biopsy if possible)
Mnemonic: C = consider cytoreduction
1. Cytopenia(s): ANC < 1000/μL or Hb < 10 g/dL or PLT < 100 000/μL
2. Hepatomegaly with ascites and impaired liver function
3. Palpable splenomegaly with associated hypersplenism
4. Malabsorption with hypoalbuminemia and weight loss
5. Skeletal lesions: Large‐sized osteolyses with pathologic fractures
6. Life‐threatening organ damage in other organ systems that is caused by local MC infiltration in tissues

Abbreviations: ANC, absolute neutrophil count; BM, bone marrow; ISM, indolent SM; MC, mast cell(s); MDS, myelodysplastic syndrome; MPN, myeloproliferative neoplasm; PLT, platelets; WHO, World Health Organization.

a In SM patients in whom less than two B‐findings and no C‐finding are detected (category A), the diagnosis indolent SM can be established. When two or more B‐findings but no C‐findings are present, the diagnosis is smouldering SM; and when 1 or more C‐findings (+/− B‐findings) are detected, the final diagnosis is either aggressive SM (<20% MCs in BM smears) or MC leukaemia (MCs ≥20% on marrow smears) (see also Table 1B).

In veterinary oncology, the most frequent clinical presentation of MC neoplasms in dogs is a solitary cutaneous nodule (Figure 1).^5^, ^6^, ^7^ MCTs can have a very heterogeneous appearance, not always accompanied by a positive Darier's sign, and the diagnosis of MCT can usually be established by a cytological examination of a fine‐needle aspiration.^5^, ^6^, ^7^ However, fine‐needle biopsy‐derived cytology is not sufficient to determine the grade of the MCT. Therefore, an additional histological examination of the lesion is mandatory to determine the grade of the disease and thus to anticipate the behavior of the MCT in dogs. A number of clinical, molecular, and histopathological variables are considered to be of prognostic significance in canine MCTs (Table S1). The first grading system was established by Patnaik et al^57^ in 1984 (Table S2). This classification system divides canine cutaneous MCTs into three grades, namely, grade 1 with well‐differentiated morphology, grade 2 with intermediately differentiated cells, and grade 3 with poorly differentiated MCs.^57^ Although this grading system correlates with the clinical outcome of patients, its practical application showed some inconsistencies because of inter‐observer variations.^58^, ^62^, ^63^ Therefore, a 2‐tier histopathological grading system has been proposed by Kiupel et al^58^ in 2011 (Table S3), with the aim to improve the Patnaik system regarding prognostication of patients. Nonetheless, it has been shown that approximately 15% to 20% of dogs with Kiupel low‐grade MCTs have metastatic disease at presentation,^64^ suggesting that there is still a need for better prognostication and an improved grading system in cutaneous MCTs, and for the moment, many centres are using both the Patnaik and Kiupel prognostication model in individual canine MCT patients. In the future, a refined prognostic grading system that includes laboratory and molecular parameters may be developed in canine MCTs. Whether *KIT* mutations and/or elevated tryptase levels are of prognostic significance in canine MCTs, remains at present unknown.

## 4 DETECTION OF KIT EXPRESSION AND EVALUATION OF THE *KIT* MUTATIONAL STATUS

In the human system, KIT is employed as a surface marker to detect normal and neoplastic MCs (KIT+/CD34‐) by flow cytometry.^2^, ^8^, ^49^, ^50^, ^51^, ^52^, ^61^ In addition, KIT is employed together with tryptase to detect and enumerate neoplastic MCs in the BM of patients with SM by immunohistochemistry.^65^, ^66^ In adult patients with SM, the activating *KIT* mutation D816V is detected in a vast majority of cases. Using highly sensitive polymerase chain reaction techniques, *KIT* D816V can be detected in the BM and in the peripheral blood of most adult human patients with SM.^67^, ^68^, ^69^ Therefore, *KIT* D816V not only serves as a disease‐related criterion but can also be employed as a PB screening biomarker for patients with suspected SM.^67^ However, in a smaller subset of patients with SM, other *KIT* mutations (in codons other than 816) or no *KIT* mutations are found.^62^ In these patients, sequencing studies of the whole *KIT* structure are sometimes recommended.^67^ However, these studies are laborious and therefore are not regarded as standard practice.

In dogs, immunohistochemical staining for KIT is of prognostic value, as different staining patterns correlate with recurrence‐rate and survival in MCT.^59^ In contrast to the human system, a number of different *KIT* mutations are detectable in dogs with MCT.^18^, ^19^, ^20^ Therefore, a more detailed evaluation of the *KIT* gene may be clinically helpful. A full sequencing profile of the *KIT* gene is not standard in daily veterinary practice. However, screening for a limited panel of *KIT* mutations known to be clinically relevant (activating) and to occur recurrently (in exon 8, 9, and 11 of *KIT*) in MCTs is recommended when the assay is available.^60^, ^70^, ^71^

## 5 STAGING INVESTIGATIONS IN PATIENTS WITH MC NEOPLASMS

In the human system, involvement of the BM is always documented by analysing BM biopsy samples by histomorphological and immunohistochemical studies and BM aspirate samples by cytomorphological, flow cytometric, cytogenetic and molecular studies.^2^, ^49^, ^50^, ^51^, ^52^, ^53^, ^54^, ^61^, ^65^, ^66^, ^72^ Standard immunohistochemical markers applied for the detection and enumeration of MCs in the BM (or other organs) are KIT (CD117) and tryptase (Figure 1). Other organ systems are usually not examined by histological studies, unless the aetiology of organopathy/organomegaly remains uncertain or the patient is suffering from a sarcoma‐like disease (MC sarcoma). However, in all patients, the size of the liver and spleen is determined by ultrasound or computed tomography (CT).^61^ In addition, the sizes of the involved lymph nodes, when enlarged, are measured by ultrasound and/or CT. Bone involvement with osteopenia or osteoporosis should be determined by osteodensitometry in all cases with SM.^61^ Osteopenia is quite frequently detected in human patients with SM, and if not treated appropriately most of these patients progress to osteoporosis that may be complicated by pathologic bone fractures.^2^, ^61^ Osteopenia/osteoporosis can occur in any form/variant of SM. By contrast, large osteolyses are rarely seen in SM and are confined to patients with advanced MC disease.^2^, ^61^ In case of suspected focal lesions and in advanced SM, an x‐ray of all bones is usually recommended. Finally, the skin is examined in detail by inspection (and by photography if possible) in all patients.^11^, ^61^

In the canine system, clinical staging includes a complete blood count with differential counts, serum chemistry and cytological or histological and immunohistochemical studies of the primary organ site and of the secondary (cutaneous or extracutaneous, metastatic) lesion(s) (Figure 1).^57^, ^58^ Fine‐needle aspiration biopsies of regional lymph nodes (even if normal in size), abdominal ultrasound (with or without fine‐needle aspiration of liver and spleen regardless of the sonographic appearance) and thoracic radiography are usually performed.^64^, ^73^ In the majority of dogs, MCTs disseminate first into the regional lymph nodes, then to the spleen and liver, and finally into other visceral organs, whereas lung involvement is rare.^74^ In case of major blood count abnormalities and/or visceral involvement, a BM examination, including cytology (in BM smears), histology (morphologic and immunohistochemical studies) is often recommended.^75^, ^76^ A detailed investigation of the BM in all dogs with MCTs is unlikely to be clinically helpful as the vast majority of cases are presented with solitary, low to intermediate grade tumours that are locally confined and do not involve the marrow compartment. Whether BM assessment in patients presenting with systemic illness, with negative prognostic indices or for whom blood dyscrasias are present on routine complete blood count can improve the staging system and treatment recommendation, remains to be explored in future investigations. Interestingly, unlike human patients with SM, neither osteopenia/osteoporosis nor osteolyses is detected in canine patients with MCT. Additional parameters, such as immunohistochemical markers (eg, CD25), *KIT* mutations, and other molecular markers are employed successfully in the diagnosis and prognostication of MC neoplasms in the human system but so far not in the canine system. Whether characterization of such parameters can be helpful in the diagnosis (primary tumour site), grading (primary tumour and other involved organs), staging (investigations of BM, blood and/or other organs), and/or prognostication of canine MCTs remain to be determined in future studies.

## 6 UTILITY OF TRYPTASE AND OTHER DISEASE PARAMETERS DURING FOLLOW‐UP

In the human system, the basal serum tryptase level is a robust and widely used follow‐up parameter for patients with SM.^77^, ^78^, ^79^, ^80^ In particular, tryptase is a serine protease that is produced and stored almost exclusively in MCs and is secreted from resting MCs into plasma in a constitutive manner.^81^, ^82^ As a result, the basal serum tryptase level reflects the total body burden of MCs in healthy individuals (normal physiologic baseline: 1‐15 ng/mL) and in patients with mastocytosis.^82^ In contrast, patients with CM generally have basal serum tryptase levels within the normal range, clearly elevated tryptase levels (>20 ng/mL) are almost invariably found in patients with SM and therefore also serve as a minor diagnostic criterion of SM.^49^ In addition, the tryptase level is an important follow‐up parameter in SM.^61^ Likewise, whereas patients with ISM have stable tryptase levels, a steadily increasing basal serum tryptase is indicative of advanced SM and/or disease progression. Furthermore, effective therapy is usually accompanied by a decrease in the serum tryptase level.^31^, ^35^, ^36^, ^37^, ^83^

In addition, blood counts and (other) serum chemistry parameters, such as alkaline phosphatase levels, are employed in the follow‐up of human patients with SM.^61^ Furthermore, liver and spleen size (by ultrasound), lymph nodes, and the osteodensitometry (T score) are measured routinely in the follow‐up of patients with SM.^61^

In dogs, physical examination, in particular investigating the initial location(s) of the MCTs and regional lymph nodes, determination of blood counts and serum chemistry parameters, and abdominal ultrasound, are standard in the follow‐up of MCT patients.^75^ At present, no MC‐specific serum‐ or plasma markers are available that could be employed as follow‐up parameters for dogs with MCTs. Whether the inclusion of serologic MC‐related follow‐up parameters (like serum tryptase) would be of value for determining the course of canine MCT and for evaluation of responses to conventional or novel therapies remains to be determined in future studies.

### 6.1 Treatment of Advanced SM and selection of anti‐neoplastic drugs

In human patients with advanced MC neoplasm with slow progression, interferon‐alpha (IFN‐A) or cladribine (2CdA) have been considered as first‐line therapy with response rates ranging between 10% and 30%.^83^, ^84^, ^85^, ^86^ In patients with rapidly progressing ASM and MCL, more intensive therapy is required. In patients who are fit and eligible, polychemotherapy containing fludarabine or 2CdA, often in combination with cytosine arabinoside (ARA‐C), are recommended, and in case of a sufficient response (clear cytoreduction), haematopoietic stem‐cell transplantation (SCT) should be considered.^37^, ^49^, ^87^, ^88^ In patients with poor performance status, 2CdA or hydroxyurea can be used as palliate drugs. A more recent approach is to apply targeted drugs directed against *KIT* D816V.^34^, ^35^, ^36^, ^37^ Midostaurin has recently been described to exert major disease‐modifying activity in patients with advanced SM including MCL.^35^, ^37^ In addition, midostaurin is useful for cytoreducing tumour load in patients with advanced SM prior to SCT.^37^ In patients with MC sarcoma, the treatment recommendation is similar to that in MCL because most of these patients transform to ASM or MCL within weeks or months.^37^, ^49^, ^87^ In addition to chemotherapy, radiation therapy may be applied in MCS patients.^37^, ^49^, ^87^ However, most patients with MCS die within a short time‐interval despite intensive therapy.

In dogs with resectable MCTs without distant metastasis, surgical intervention is the first‐line therapy, especially for solitary grade I MCTs.^7^ In cases of incomplete resection (“dirty margins”), re‐excision is recommended whenever possible.^89^ If re‐excision is considered impossible because of diffuse infiltration or massive tumour expansion, post‐surgical radiation therapy is required, and in high‐grade and/or metastasized MCT, radiation is usually combined with systemic anti‐neoplastic therapy.^90^, ^91^ In dogs with high‐grade metastasized or unresectable MCTs, first‐line treatment often consists of TKI therapy using masitinib (EU), toceranib (US, EU) or imatinib (Japan) or cytoreductive chemotherapeutics (eg, vinblastine, lomustine; often in combination with steroids).^38^, ^39^, ^40^ More recently, the combination of toceranib with radiation therapy has been described to be an effective anti‐neoplastic treatment approach for dogs with MCTs, including high‐grade MCTs.^92^ In case of primary or secondary resistance, different chemotherapy protocols using alternative cytoreductive drugs or combinations with TKI are prescribed.^93^, ^94^, ^95^, ^96^, ^97^, ^98^, ^99^ However, although remissions can be achieved, responses are usually short‐lived.^93^, ^94^, ^95^, ^96^, ^97^, ^98^, ^99^ A summary of interventional treatment approaches in advanced SM in the human and canine system is depicted in Table 4.

**Table 4:** Standard interventional therapies in advanced MC neoplasms in human and dogs

	Development status and indication in	
Drug/therapy	Human patients	Canine patients
Imatinib	Approved for the treatment of aggressive SM without D816V KIT mutant or with unknown *KIT* mutation status	Used off‐label for therapy of advanced MC tumours in Japan
Cladribine (2CdA)	Orphan drug approval	n.a.
Midostaurin	Approved for the treatment of advanced, including ASM and MCL	n.a.
Toceranib		Approved for therapy of canine non resectable Patnaik grade 2/3 MCTs (by EMA and FDA)
Masitinib	Advanced SM without KIT D816V +/− severe mediator symptoms	Approved for therapy of canine non resectable Patnaik grade 2/3 MCTs with *KIT* mutations (by EMA)
Polychemotherapy	CT like in AML	VBL/Pred; CCNU/Pred, also in combination with TKI
SCT	Standard in ASM‐t and MCL after debulking in young and fit patients	Not available in daily practice

Abbreviations: ASM, aggressive systemic mastocytosis; CCNU, chlorethyl‐cyclohexyl‐nitroso‐urea, lomustine; CT like in AML, chemotherapy like in acute myelocytic leukaemia; EMA, European Medicines Agency; FDA, Food and Drug Administration; MC, mast cell; MCL, mast cell leukaemia; n.a., not analysed; Pred., prednisolone; SCT, haematopoietic stem‐cell transplantation; SM, systemic mastocytosis; TKI, tyrosine kinase inhibitor; VBL, vinblastine.

Concomitant treatment with H1‐ and H2‐blockers is recommended to prevent mediator‐related side effects in all patients.^100^, ^101^ Whether the use of proton‐pump inhibitors may improve the symptomatic treatment effect in canine MCT patients like in human SM patients remained to be evaluated in future clinical trials.^61^

### 6.2 Concluding remarks and future perspectives

Comparative oncology is an emerging field that supports the development of new diagnostic and therapeutic concepts in various tumour models. One example highlighted in this report is comparative research in human and canine MC neoplasms. In both species, indolent and aggressive disease variants have been described and in both species, advanced MC neoplasms are often treated by cytoreductive drugs and KIT‐targeting TKI. However, in both species, there is still a need to develop improved diagnostic criteria, improved prognostication models, and better (curative) drug therapies. We strongly believe that comparative oncology approaches may support these developments. Likewise, based on the higher frequency of canine MCTs, *in vivo* studies with various drugs and drug combinations may be more feasible in dogs with high grade and/or metastasized MCTs than in human patients with advanced SM. On the other hand, diagnostic and prognostic parameters developed into SM criteria in the human system such as the *KIT* mutational status, the aberrant expression of surface markers (CD2, CD25, and/or CD30) or the serum tryptase level, should be tested for their diagnostic and/or prognostic value in canine MCT. Whether comparative studies will indeed lead to improved prognostication and therapy in canine and human patients with MC neoplasms remains to be determined in forthcoming investigations. Such comparative strategies require an interdisciplinary dialogue between human and veterinary medicine.

## Supporting information


**Appendix S1.** Supporting information.
**Table S1**. Prognostic markers for canine mast cell neoplasms.
**Table S2**. Patnaik morphologic grading classification for canine cutaneous mast cell tumours (1984).^1^
**Table S3**. Kiupel two‐tier grading criteria for canine cutaneous mast cell tumours (2011).^2^
