# Supplementary material for: Comparative oncology: The paradigmatic example of canine and human mast cell neoplasms
Source: Vet Comp Oncol. 2018 Sep 24;17(1):1–10. doi: 10.1111/vco.12440 (PMC6378619; doi:10.1111/vco.12440)
Supplement: Supplementary file 1 — Appendix S1. Supporting information. Table S1. Prognostic markers for canine mast cell neoplasms. Table S2. Patnaik morphologic grading classification for canine cutaneous mast cell tumours (1984).1 Table S3. Kiupel two‐tier grading criteria for canine cutaneous mast cell tumours (2011).2 [file VCO-17-1-s001.docx]

Supplemental Data to the Manuscript

**Comparative Oncology: the Paradigmatic Example of Canine**

**and Human Mast Cell Neoplasms**

**Supplementary Table S1**

Prognostic Markers for Canine Mast Cell Neoplasms

-------------------------------------------------------------------------------------------------------------

References

-------------------------------------------------------------------------------------------------------------

Histologic Grade Patnaik^1^, Kiupel^2^, Thamm^3^, Webster^4^, Giantin^5^, Sledge^6^

Clinical stage Krick^7^, Warland^8^, Worley^9^, Murphy^10^, Hillman^11^, Horta^12^

Proliferation Marker

Mitotic Index Kiupel^2^, Horta^12^, Thompson^13^

Ki67 Webster^4^, Horta^12^, Scase^14^, Abadie^15^

AgNOR Thamm^3^, Webster^4^, Scase^5^

IHC KIT pattern Giantin^5^, Horta^12^, Kiupel^16^

*KIT* Mutation Webster^4^, Giantin^5^, Sledge^6^, Horta^12^, Takeuchi^17^, London^18^

Others

Response to TKI treatment Horta^19^

--------------------------------------------------------------------------------------------------------------

Ki67, Ki67 nuclear protein; IHC KIT pattern, immunohistochemical KIT pattern; AgNOR, Argyrophilic Nucleolar organizer regions.

**Supplementary Table S2**

Patnaik Morphologic Grading Classification for Canine Cutaneous MC Tumours (1984)^1^

-----------------------------------------------------------------------------------------------------------------

Tumor Grade

-----------------------------------------------------------------------------------------------------------------

I II III

-----------------------------------------------------------------------------------------------------------------

Location Dermis and interfollicular Infiltrate lower dermal and Replace subcutaneous

Spaces subcutaneous tissue; some and deep tissues

extend to skeletal muscles

or surrounding tissues

Cell morphology Round, monomorphic, Round to ovoid, moderately Round, ovoid, spindle

ample distinct cytoplasm pleomorphic, with scattered shaped, pleomorphic,

with medium-sized spindle and giant cells; most medium sized; cytoplasm

granules cells distinct cytoplasm with indistinct with granules

fine granules, but some with that are fine or not

indistinct cytoplasm and obvious; many giant cells

large/hyperchromic granules and scattered multi-

nucleated cells

Nuclear Round, condensed Round to indented with Indented to round

morphology chromatin scattered chromatin and vesiculated, with 1 or

single nucleoli; some with more prominent

double nuclei nucleoli; common bi-

nucleated cells

Architecture, Arranged in rows or Moderately to highly cellular; Cellular, arranged in

Cellularity, stromal small groups, separated arranged in groups with thin closely packed sheets;

Reaction by mature collagen fibrovascular stroma (some- stroma fibrovascular or

fibers of the dermis times thick and fibro- thick and fibrocollagenous

collagenous with areas of with areas of hyalinization

hyalinization)

Mitotic figures None Rare (0-2/HPF) Common (3-6/HPF)

Edema and necrosis Minimal Areas of diffuse edema Edema, hemorrhage, and and necrosis and necrosis common

----------------------------------------------------------------------------------------------------------------------------------------HPF, high-power field

**Supplementary Table S3**

Kiupel Two-Tier Grading Criteria for Canine Cutaneous MC Tumours (2011)^2^

----------------------------------------------------------------------------------------------------------------------------------------

Tumor Grade

------------------------------------------------------------------------------------------------------

low grade high grade

----------------------------------------------------------------------------------------------------------------------------------------

Mitotic figures < 7 MF/10 HPF > 7 MF/10 HPF

Cell Morphology < 3 multinucleated cells /10 HPF > 3 multinucleated cells /10 HPF

Nulear Morphology < 3 bizarre nuclei/10 HPF > 3 bizarre nuclei/10 HPF

Karyomegaly < 10% of neoplastic cells > 10% of neoplastic cells vary by 2-fold

----------------------------------------------------------------------------------------------------------------------------------------

MF, mitotic figures; HPF, high-power fields

**References**

1. Patnaik AK, Ehler WJ, MacEwen EG. Canine cutaneous mast cell tumor: morphologic grading and survival time in 83 dogs. *Veterinary Patholology* 1984; **21**: 469-474.

2. Kiupel M, Webster JD, Bailey KL, Best S, DeLay J, Detrisac CJ, et al. Proposal of a 2-tier histologic grading system for canine cutaneous mast cell tumors to more accurately predict biological behavior. *Veterinary Pathology* 2011; **48**: 147-155.

3. Thamm DH, Turek MM, Vail DM. Outcome and prognostic factors following adjuvant prednisone/vinblastine chemotherapy for high-risk canine mast cell tumour: 61cases. *Journal of Veterinary Medical Science* 2006; **68**: 581-587.

4. Webster JD, Yuzbasiyan-Gurkan V, Thamm DH, Hamilton E, Kiupel M. Evaluation of prognostic markers for canine mast cell tumors treated with vinblastine and prednisone. *BMC Veterinary Research* 2008; **4**: 32.

5. Giantin M, Vascellari M, Morello EM, Capello K, Vercelli A, Granato A, et al. c-KIT messenger RNA and protein expression and mutations in canine cutaneous mast cell tumors: correlations with post-surgical prognosis. *Journal of Veterinary Diagnostic Investigation* 2012; **24**:116-126.

6. Sledge DG, Webster J, Kiupel M. Canine cutaneous mast cell tumors: A combined clinical and pathologic approach to diagnosis, prognosis, and treatment selection. *The Veterinary Journal* 2016; **215**: 43-54.

7. Krick EL, Kiupel M, Durham AC, Thaiwong T, Brown DC, Sorenmo KU. Investigating Associations Between Proliferation Indices, C-kit, and Lymph Node Stage in Canine Mast Cell Tumors. *Journal of the American Animal Hospital Association* 2017; **53**: 258-264.

8. Warland J, Amores-Fuster I, Newbury W, Brearley M, Dobson J. The utility of staging in canine mast cell tumours. *Journal of Veterinary and Comparative Oncology* 2014; **12**: 287-298.

9. Worley DR. Incorporation of sentinel lymph node mapping in dogs with mast cell tumours: 20 consecutive procedures. *Journal of Veterinary and Comparative Oncology* 2014; **12**: 215-226.

10. Murphy S, Sparkes AH, Blunden AS, Brearley MJ, Smith KC. Effects of stage and number of tumours on prognosis of dogs with cutaneous mast cell tumours. *Veterinary Record* 2006; **158**: 287-291.

11. Hillman LA, Garrett LD, de Lorimier LP, Charney SC, Borst LB, Fan TM. Biological behavior of oral and perioral mast cell tumors in dogs: 44 cases (1996-2006). *Journal of the American Animal Hospital Association*. 2010; **237**: 936-942.

12. Horta RS, Lavalle GE, Monteiro LN, Souza MCC, Cassali GD, Araújo RB. Assessment of canine mast cell tumor mortality risk based on clinical, histologic, immunohistochemical, and molecular features. *Veterinary Pathology* 2018: 300985817747325.

13. Thompson JJ, Pearl DL, Yager JA, Best SJ, Coomber BL, Foster RA. Canine subcutaneous mast cell tumor: characterization and prognostic indices. *Veterinary Patholology* 2011; **48**: 156-168.

14. Scase TJ, Edwards D, Miller J, Henley W, Smith K, Blunden A, et al. Canine mast cell tumors: correlation of apoptosis and proliferation markers with prognosis. *Journal of Veterinary Internal Medicine* 2006; **20**:151-158.

15. Abadie JJ, Amardeilh MA, Delverdier ME. Immunohistochemical detection of proliferating cell nuclear antigen and Ki-67 in mast cell tumors from dogs. *Journal of the American Veterinary Medical Association* 1999; **215**: 1629-1634.

16. Kiupel M, Webster JD, Kaneene JB, Miller R, Yuzbasiyan-Gurkan V. The use of KIT and tryptase expression patterns as prognostic tools for canine cutaneous mast cell tumors. *Veterinary Patholology* 2004; **41**: 371-377.

17. Takeuchi Y, Fujino Y, Watanabe M, Takahashi M, Nakagawa T, Takeuchi A, et al. Validation of the prognostic value of histopathological grading or c-kit mutation in canine cutaneous mast cell tumours: a retrospective cohort study. *The Veterinary Journal* 2013; **196**: 492-498.

18. London CA, Malpas PB, Wood-Follis SL, Boucher JF, Rusk AW, Rosenberg MP, et al. Multi-center, placebo-controlled, double-blind, randomized study of oral toceranib phosphate (SU11654), a receptor tyrosine kinase inhibitor, for the treatment of dogs with recurrent (either local or distant) mast cell tumor following surgical excision. *Clinical Cancer Research* 2009; **15**: 3856-3865.

19. Horta RDS, Giuliano A, Lavalle GE, Costa MP, de Araújo RB, Constantino-Casas F, et al. Clinical, histological, immunohistochemical and genetic factors associated with measurable response of high-risk canine mast cell tumours to tyrosine kinase inhibitors. *Oncology Letters* 2018; **15**:129-136.
